# Achieving Low Dissipation Factors and Low Dielectric Constants via Thermally Stable Naphthalene-Based Poly(ester-imide)s with Fluorine Groups

**DOI:** 10.1021/acsami.5c00599

**Published:** 2025-03-13

**Authors:** Manohar Reddy Busireddy, Ling-Huan Meng, Jin-Wei Lin, Wei-Chung Ke, Jiun-Tai Chen, Chain-Shu Hsu

**Affiliations:** † Department of Applied Chemistry, National Yang Ming Chiao Tung University, 1001 University Road, Hsinchu 300093, Taiwan; ‡ Center for Emergent Functional Matter Science, National Yang Ming Chiao Tung University, 1001 University Road, Hsinchu 300093, Taiwan

**Keywords:** poly(ester-imide)s, naphthalene, trifluoromethyl groups, high thermal stability, molar free volume, low dielectric constant, ultralow dissipation factor

## Abstract

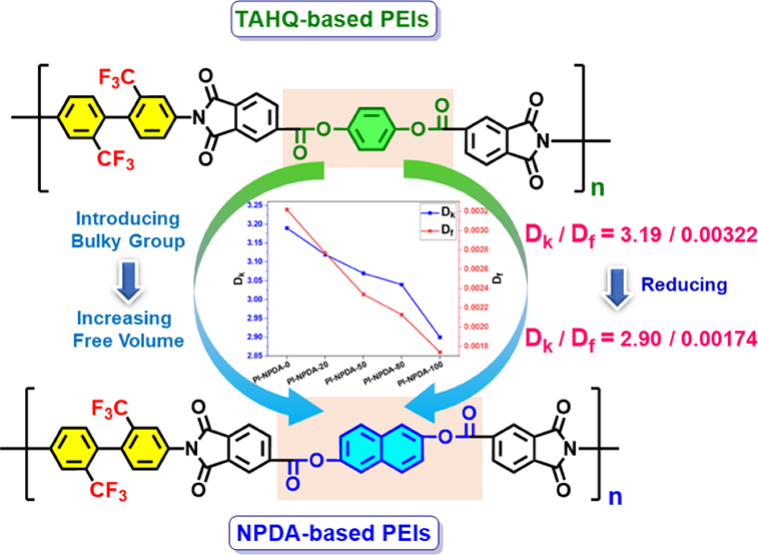

In recent years, polyimides (PIs) and their functional derivatives (i.e., poly(ester-imide)s (PEIs), poly(amide-imide)s (PAIs), etc.) have gained more attention in the microelectronic and optoelectronic industries because of their excellent thermal-mechanical stabilities, heat resistance, dielectric properties, and solution processabilities. Nevertheless, developing PIs with low dielectric constants (*D*_k_) and low dissipation factors (*D*_f_) at high frequencies (≥10 GHz) is crucial for advanced mobile communications and high-frequency applications. Therefore, to achieve low dielectric properties in both *D*_k_ and *D*_f_ at high frequencies, using PEIs with strong electron-withdrawing fluorine groups is a good synthetic strategy. Herein, a series of PEIs with trifluoromethyl (−CF_3_) groups are prepared from 2,2′-bis(trifluoromethyl)-[1,1′-biphenyl]-4,4′-diamine (TFMB), 1,4-phenylene bis(1,3-dioxo-1,3-dihydroisobenzofuran-5-carboxylate) (TAHQ), and naphthalene-2,6-diyl bis(1,3-dioxo-1,3-dihydroisobenzofuran-5-carboxylate) (NPDA) monomers. In addition, the molar ratio of TAHQ/NPDA modulates the comprehensive performances of the derived PEI films, and each performance of the PEI films is addressed in detail. From the theoretical calculations, when increasing the NPDA ratio from 0 to 100% in the TFMB/TAHQ system, the molar free volume increases and balances the polarizability and linearity, which is beneficial for achieving low dielectric properties. As a result, all the PEI films exhibit excellent thermal stabilities with thermal decomposition temperature at 5% weight loss (*T*_d5%_) over 470 °C and glass transition temperature (*T*_g_) at 215–250 °C. In addition, all the PEI films show decent coefficients of thermal expansion (CTE) in the range of 16–40 ppm/°C and good mechanical stabilities. Moreover, all PEI films exhibit low dielectric properties at high frequencies. When increasing the NPDA concentration from 0 to 100%, both *D*_k_/*D*_f_ values gradually decrease from 3.19/0.00322 for PEI-NPDA-0 to 2.90/0.00174 for PEI-NPDA-100 at a 10 GHz frequency. Particularly, the *D*_f_ values are one of the lowest values under the 10 GHz frequency. The results demonstrate that preparing PEI backbones with fluorine groups is a good synthetic strategy for achieving low dielectric properties at higher frequencies.

## Introduction

The rapid development of 5G communication technologies and the Internet of Things (IoT) calls for high-performance polymers with low dielectric constants (*D*_k_) and low dielectric dissipation factors (*D*_f_) at high operating frequencies (GHz).^1−4^ Recently, a wide variety of low dielectric polymers with low *D*_k_ and *D*_f_ at high frequencies have been reported, such as polyimides (PIs) and their derivatives (i.e., poly(ester-imide)s (PEIs), poly(amide-imide)s (PAIs), etc.), liquid crystalline polymers, benzocyclobutene resins, poly(phenyleneether), fluoropolymers, polytetrafluoroethylene, polynaphthalene, etc.^5−19^ Despite all, PIs have gained more attention due to their high thermal stabilities and mechanical strengths, wide temperature resistances, good film-forming properties, high insulating performances, low dielectric properties, and promising applications in fabricating flexible printed circuit boards. Therefore, PI films are widely used in smartphones, aerospace, new energy vehicles, and other microelectronic applications.^20−28^ With the advancement of technologies, however, the dielectric properties of conventional PI films are no longer able to meet the demands of high-frequency applications (*D*_k_ ≤ 3.2; *D*_f_ ≤ 0.004).^10,20^ For instance, commercial Kapton films show *D*_k_ of 3.2 and *D*_f_ of 0.008 at 1 GHz.^20,29^ Generally, the signal transmission loss (α) of the electronic equipment strongly depends on low *D*_k_ and *D*_f_ values of the used polymers, as elucidated by the modified Maxwell’s eq 1.^10,30,31^ The α value is inversely proportional to the square root of the *D*_k_, *D*_f_, and the working frequency (*f*), as shown in eq 1.
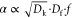
1
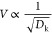
2

As demonstrated in eqs 1 and 2, PI films with low *D*_k_ and *D*_f_ are beneficial for reducing signal loss (α) and enhancing transmission speed (*V*) in the signal transmission process at high frequencies (*f*).^9,10^ Therefore, developing new PIs with low *D*_k_ and *D*_f_ at higher frequencies is essential for high-speed wireless IoT technologies.

In recent years, many researchers have proposed various approaches to achieve low *D*_k_ properties in PIs by using the well-known Clausius–Mossotti equation, *D*_k_ = (1 + 2*P*/*V*)/(1 – *P*/*V*), where “*P*” is the molar polarization and “*V*” is the molar volume.^9,11,16^ According to the Clausius–Mossotti equation, the structure-dielectric relationship of PI films mainly depends on the molar polarizability and the free volume of groups in the PI structure, which leads to reducing the *D*_k_ value.^9−11^ Consequently, reducing both *D*_k_ and *D*_f_ values can be influenced by three key factors: 1) decreasing the polarizability by introducing low polarizing groups (e.g., trifluoromethane (−CF_3_) groups and fluorine (F) atoms),^32−34^ 2) increasing the free volume of the PIs by introducing bulky groups or porous structures,^21,34−39^ and 3) reducing the dipole moment of the PI backbone by substituting polar groups.^40,41^ From the previous reports, poly(ester-imide)s (PEIs) are well-known for their ultralow *D*_f_ values at high frequencies.^9,11,16,42,43^ For instance, introducing 1,4-phenylene diester functionalized groups into the PIs can achieve an ultralow *D*_f_ value because ester-functionalized groups can enhance the coplanarity/rigidity in the PI backbone.^9,16^ Recently, Zhang et al. reported a series of linear-backbone containing PIs with ether and ester functionalized groups.^42^ The resulting PEIs show ultralow *D*_f_ values; however, a little higher *D*_k_ values were achieved because the *D*_k_ of PIs is strongly correlated with the volume polarization (α/*V*) and the free volume. Both low *D*_k_ and low *D*_f_ values can be achieved by controlling the polarization and free volume. Recently, Chen and coworkers reported several types of PIs with the structure-dielectric relationship of PIs.^9,11,16^ As a result, ether/fluorine- and ester/fluorine-attached PIs effectively reduce both *D*_k_ and *D*_f_ values. Therefore, for achieving low dielectric values at higher frequencies, preparing the PI/PEI backbone with fluorine groups is a good synthetic strategy.

**Figure 1:**
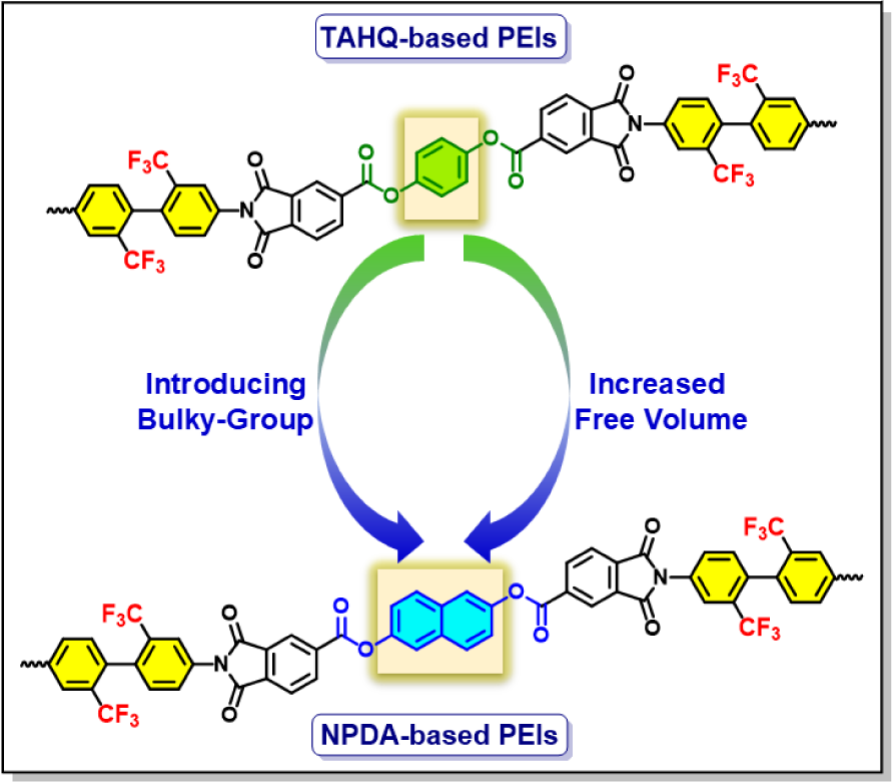
Illustration of the chemical structure-dielectric designing approach of PEIs.

Therefore, to reduce both *D*_k_ and *D*_f_ values, herein, we select -CF_3_ group substituted diamine and bulky backbone with ester-functionalized anhydride monomers to prepare a series of PEIs. Initially, as demonstrated in Figure 1, a 2,2′-bis(trifluoromethyl)-[1,1′-biphenyl]-4,4′-diamine (TFMB) and 1,4-phenylene bis(1,3-dioxo-1,3-dihydroisobenzofuran-5-carboxylate) (TAHQ)-based pure TFMB/TAHQ PEI film is fabricated. Subsequently, bulky naphthalene-core containing naphthalene-2,6-diyl bis(1,3-dioxo-1,3-dihydroisobenzofuran-5-carboxylate) (NPDA) monomer is gradually increased in the TFMB/TAHQ system, and a series of Co-PEI and pure TFMB/NPDA PEI films are prepared. The obtained PEIs are named PEI-NPDA-0, PEI-NPDA-20, PEI-NPDA-50, PEI-NPDA-80, and PEI-NPDA-100 for NPDA compositions of 0, 20, 50, 80, and 100%, respectively. When the NPDA concentration (from 0 to 100%) is increased in the TFMB/TAHQ system, the values of molar volume and fractional free volume (FFV) are gradually increased, which are calculated from theoretical calculations. As a result, both *D*_k_ and *D*_f_ values are reduced gradually from 3.19/0.00322 for PEI-NPDA-0 to 2.90/0.00174 for PEI-NPDA-100 at 10 GHz frequency. Furthermore, all of the PEI films exhibit high thermal stabilities and mechanical strengths. From these results, preparing the fluorine group-substituted bulky backbone with ester-functionalized PEIs is a good synthetic strategy for achieving both low *D*_k_ and *D*_f_ at higher frequencies.

## Experimental Section

### Materials

The utilized materials of this work are demonstrated as follows: 2,6-dihydroxynaphthalene (98%) and trimellitic anhydride chloride (TMC, 95%) were received from Nova Materials. Pyridine (99%) and acetic anhydride (100%) were purchased from Thermo Scientific and Honeywell Fluka, respectively. *N*-Methyl-2-pyrrolidone (NMP) was purchased from Acros Organics and stored under a nitrogen (N_2_) atmosphere with 4 Å molecular sieves. Dry tetrahydrofuran (THF) was purchased from Echo Chemical, and deuterated dimethyl sulfoxide (DMSO-*d*_6_) was received from Sigma-Aldrich. 2,2′-Bis(trifluoromethyl)-[1,1′-biphenyl]-4,4′-diamine (TFMB) and 1,4-phenylene bis(1,3-dioxo-1,3-dihydroisobenzofuran-5-carboxylate) (TAHQ) were received from Shifeng Technology with 99%+ purity. These chemicals and solvents were used without further purification to construct the PEI film in this study.

### Measurements

Inherent viscosities were measured in NMP at a concentration of 0.5 g/dL at 30 °C by using an Ubbelohde viscometer. A JEOL 400 MHz nuclear magnetic resonance (NMR) spectrometer was used to measure the ^1^H and ^13^C NMR spectra of the NPDA monomer. A JEOL instrument (model: JMS-T200GC AccuTOF GCx; source mode: FD (field desorption)) was conducted to measure the FD mass spectral data of the NPDA monomer. Attenuated total reflectance Fourier-transform infrared (ATR-FT-IR) spectroscopy data of the PEI films were obtained from a Spectrum One (PerkinElmer) instrument. The mechanical properties of the PEI films were measured using a Shimadzu EZ-L instrument following the ASTM D882 guideline. The films [100 mm (*L*) × 10 mm (*W*) × 40 ± 2 μm (*T*)] were stretched at a drawing rate of 12.5 mm min^–1^. To measure the coefficient of thermal expansion (CTE) values of all PEI films, a TA Instrument TMA450EM was used at a heating rate of 5 °C min^–1^, and the CTE values were calculated from 100 to 200 °C in the second heating cycle. The decomposition temperature (*T*_d5%_) data were collected from a TA Instruments TGA55 at a rate of 20 °C min^–1^ up to 700 °C. The glass transition temperatures (*T*_g_s) were measured via a TA Instruments DMA850 at a rate of 3 °C min^–1^ up to 260 °C and with a load frequency (sinusoidal) of 1.0 Hz in air, and the *T*_g_ values were calculated from peaks of the tan δ (E′/E″) curves. The dielectric properties (*D*_k_ and *D*_f_) were determined by a high-frequency vector network analyzer (Anritsu MS46122B) with a kit of 10 GHz cavity D2014-1; the operating condition was managed at 22 °C and 50% humidity. A contact angle analyzer (First Ten Ångstrom, FTA125) was used to examine the static contact angles of the PEI films with water. A water absorption test was conducted on the PEI films. In this process, all the PEI films were immersed in deionized water at 25 °C, and the weight differences of all the films were measured after 24 h. Fractional free volume (FFV) was calculated as follows: FFV = (*V* – *V*_0_)/*V*, *V*_0_ = 1.3 *V*_w_, “*V*” is the molar volume of the repeating unit (cm^3^ mol^–1^), “*V*_0_” is the volume occupied by the molecular chain (cm^3^ mol^–1^), and “*V*_w_” is the van der Waals volume, which was calculated via the group contribution Bondi method. “*V*” is calculated from the formula: *d* (density) = *M*/*V*. The densities of the films were measured via a Mettler-Toledo balance ME204T coupled with a density kit utilizing Archimedes’ principle. The optimized structures, molar volumes, and dipole moments of TFMB/TAHQ and TFMB/NPDA were accomplished via utilizing DFT calculation with the B3LYP/6-311G(d,p) basis set. Jasco gel permeation chromatography (GPC) analysis was conducted to characterize the NPDA-based PAA solutions using polystyrene as the standard and dimethylformamide as the eluent at 25 °C. Scanning electron microscopy (SEM) images were captured by a field emission scanning electron microscope (JEOL, JSM-7401F). Atomic force microscopy (AFM) images were obtained on a BrukerDMFASTSCAN2-SYS instrument in tapping mode.

### Synthesis of Naphthalene-2,6-diyl Bis(1,3-dioxo-1,3-dihydroisobenzofuran-5-carboxylate) (NPDA) Monomers

In a 250 mL round-bottom flask, 2,6-dihydroxynaphthalene (5 g, 31.22 mmol) was dissolved in 25 mL of THF and stirred under nitrogen protection. Next, trimellitic anhydride chloride (TMC) (19.7 g, 93.65 mmol) was dissolved in 100 mL of THF, and this solution was then added to the 2,6-dihydroxynaphthalene/THF mixture; the resulting solution mixture was stirred vigorously at 25 °C for 10 min. Thereafter, 15 mL (186 mmol) of pyridine was slowly (dropwise) added to the reaction mixture. The resulting solution was then stirred at 25 °C for 24 h. Following the completion of the reaction, the reaction mixture was filtered to collect the yellow solid precipitate and the product was repeatedly washed with acetic anhydride for 4 h. Finally, vacuum-dried at 100 °C for 18 h to obtain a yellow color solid (14.3 g, Yield = 90.11%). ^1^H NMR (400 MHz, DMSO-*d*_6_, ppm): δ 8.46 (d, *J* = 2.0 Hz, 2H), 8.38 (dd, *J* = 8.0, 1.6 Hz, 2H), 8.08 (d, *J* = 8.8 Hz, 2H), 7.99 (d, *J* = 2.4 Hz, 2H), 7.88 (d, *J* = 8.0 Hz, 2H), 7.61 (dd, *J* = 8.8, 2.4 Hz, 2H); ^13^C NMR (100 MHz, DMSO-*d*_6_, ppm): δ 168.37, 167.33, 163.63, 148.24, 138.49, 132.48, 132.41, 131.57, 130.65, 130.00, 129.29, 129.04, 122.47, 118.92; FD-MS cald. for C_28_H_12_O_10_ (M^+^) = 508.39, found = 508.0.

### Preparation of the PAA Solutions

The synthesis process of PEI-NPDA-50 poly(amic acid) (PAA) solution is taken as a typical synthesis route and is described as follows. NPDA (1.4234 g, 2.8 mmol), TAHQ (1.2833 g, 2.8 mmol), TFMB (1.7933 g, 5.6 mmol), and NMP (13.1 mL) were added to a three-neck flask equipped with a stirring device and a reflux device under an N_2_ atmosphere. Then the mixture was stirred at room temperature for 20 h to form the PEI-NPDA-50 PAA solution (where 50% is the molar percentage of monomer NPDA in the total dianhydride monomers). The total solid content of the reaction was 25 wt %.

### Preparation of the PEI Films

The synthesized NPDA-based PAA solutions were dropped on precleaned glass substrates, and a doctor blade was used to spread the solutions uniformly on the substrates. Subsequently, the casted glass substrates were placed in a hot N_2_ gas circulation oven. The thermal imidization process was conducted as follows: 70 °C for 1 h, 200 °C for 1 h, and finally 300 °C for 1 h at a heating rate of 2 °C per min. After the mixture was cooled to room temperature, all the PEI films were obtained by peeling from the glass in water. The obtained PEI films have thicknesses of about 40 μm (±2 μm), and these PEI films are not soluble in common organic solvents such as THF, DMAc, dimethyl sulfoxide, NMP, chloroform (CHCl_3_), and methanol (CH_3_OH).

## Results and Discussion

### Synthesis and Characterizations of the NPDA-Based PEI Films

**Figure 2:**
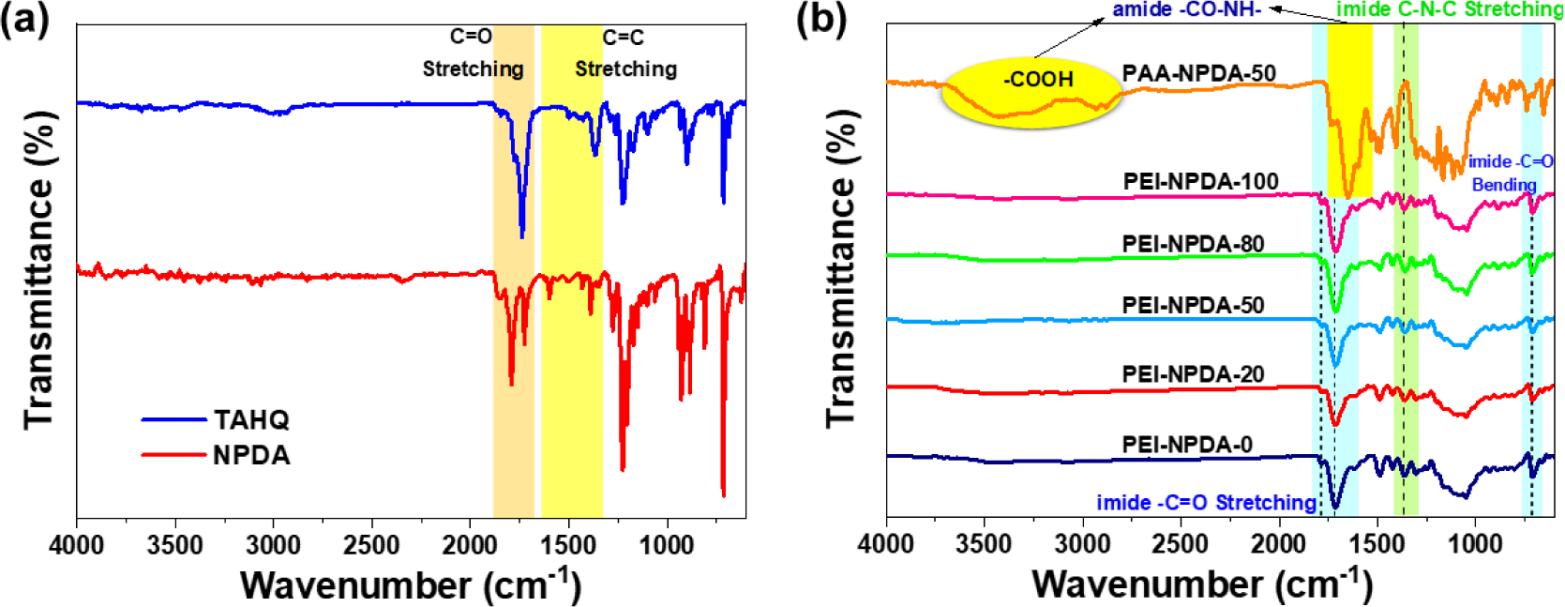
ATR-FT-IR spectra of (a) dianhydride monomers and (b) NPDA-based PEI films.

**Scheme 1:**
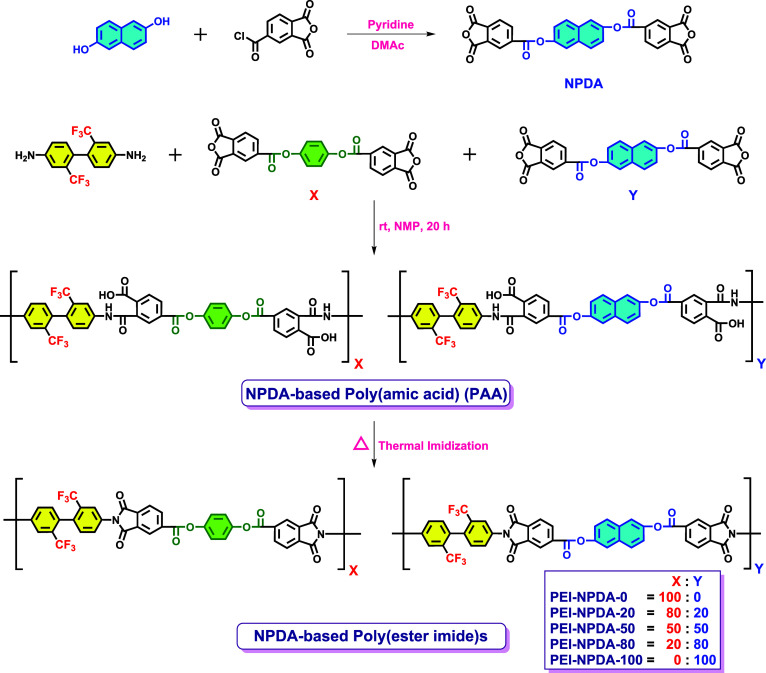
Synthetic Scheme of the NPDA-Based PEI Films

As shown in Scheme 1, naphthalene-2,6-diyl bis(1,3-dioxo-1,3-dihydroisobenzofuran-5-carboxylate) (NPDA) monomer is synthesized through an esterification reaction between naphthalene-2,6-diol and TMC in the presence of pyridine.^44^ The ^1^H NMR, ^13^C NMR, and LR-FD mass spectral data (Figures S1–S3) are presented in the Supporting Information. Additionally, an attenuated total reflectance Fourier-transform infrared (ATR-FT-IR) spectrometer is used to examine the synthesized NPDA and commercial TAHQ dianhydride monomers, and the obtained spectra are displayed in Figures 2a and S6a. The C=O and C–O stretching bands of NPDA/TAHQ are observed within the 1700–1800 and 1200–1250 cm^–1^ region, respectively. The aromatic C=C stretching bands of TAHQ are observed within 1325–1520 cm^–1^; however, the NPDA aromatic C=C stretching bands are observed within 1325–1625 cm^–1^, indicating extended conjugation.^44^

Thereafter, the NPDA-based PEIs are synthesized through a two-step polymerization method, i.e., chemical polymerization followed by thermal imidization. Initially, the poly(amic acid) (PAA) precursor is formed via the ring-opening polyaddition reaction between diamines and dianhydrides. Subsequently, thermal imidization is successively conducted to acquire the desired NPDA-based PEI films. The Jasco gel permeation chromatography (GPC) analysis is performed to measure the number-average molecular weights (*M*_n_), the weight-average molecular weight (*M*_w_), and the polydispersity index (*Đ*) of the NPDA-based PAA solutions, and the results are summarized in Table 1. In addition, all the NPDA-based PAA solution’s inherent viscosities were measured in NMP solution at a concentration of 0.5 g/dL by using an Ubbelohde viscometer at 30 °C. The calculated inherent viscosities of PEI-NPDA-0, PEI-NPDA-20, PEI-NPDA-50, PEI-NPDA-80, and PEI-NPDA-100 PAA solutions are 1.14, 0.86, 0.73, 0.76, and 0.90 dL/g, respectively, which are consistent with GPC results (Table 1).

**Table 1:** Inherent Viscosities and Molecular Weights of the NPDA-Based PAA Solutions

PAA solution	η_inh_(dL/g)^a^	*M*_n_ (×10^4^ g/mol)^b^	*M*_w_ (×10^4^ g/mol)^b^	PDI (*Đ***)**^b^
**PAA-NPDA-0**	1.14	3.9	8.4	2.15
**PAA-NPDA-20**	0.86	3.4	6.9	2.02
**PAA-NPDA-50**	0.73	2.9	6.4	2.20
**PAA-NPDA-80**	0.76	3.3	6.4	1.93
**PAA-NPDA-100**	0.90	3.6	7.0	1.94

a Inherent viscosity.

b Obtained from high temperature gel permeation chromatography (GPC) analysis [*M*_n_ = The number-average molecular weights; *M*_w_ = the weight-average molecular weight; PDI (*Đ*) = polydispersity index].

Furthermore, the NPDA-based PAA solutions and the corresponding PEI films are measured using an ATR-FT-IR spectrometer, and the results are demonstrated in Figures 2b and S6. The specific characteristic absorption bands at 2800–3700 cm^–1^ of the O–H stretching bond and at 1520–1740 cm^–1^ of the amide bonds (C=O/C–NH) are observed in the IR spectra of the PAA solution. Figure S6b shows that, as the NPDA ratio increases, different characteristic behaviors of the aromatic C=C stretching bands are observed within the 1440–1575 cm^–1^ region, indicating that copolymerization occurs. To further confirm the copolymerization, ^1^H NMR analysis is conducted for the NPDA-based PAA solutions. The resulting ^1^H NMR spectra are depicted in Figures S4–S5. As shown in Figure S4, the N–H and COOH proton peaks appear within the ranges of δ10.88–11.02 and 13.30–13.80 ppm, respectively. As demonstrated in Figure S5, when increasing the NPDA monomer concentration, the NPDA (naphthalene and anhydride ring opening benzene) proton peaks are observed within the range of δ7.55–7.65 (belongs to A), 7.95–8.05 (belongs to C), 8.35–8.45 (belongs to D), and 8.62–8.68 (belongs to E) ppm, and B and F proton peaks merge with other aromatic ring proton peaks. At the same time, TAHQ monomer peaks disappear, especially in the ranges δ7.43–7.52, 8.30–8.35, and 8.58–8.62 ppm. The results confirm that copolymerization occurs in the NPDA-based PEIs.

After thermal imidization, the complete conversion of *o*-carboxylic amide to the imide ring is confirmed by the disappearance of the absorption bands of O–H and amide stretching peaks at 2800–3700 and 1520–1740 cm^–1^, respectively. Subsequently, the characteristic absorption bands of the imide ring appear at 1784/1716 cm^–1^ (C=O asymmetry/symmetry stretching), 1367 cm^–1^ (C–N stretching), and 714 cm^–1^ (C=O bending), which indicates that the PEI films have completed the imide ring formation. In addition, different characteristic behaviors of the aromatic C=C stretching bands within the 1425–1650 cm^–1^ region are observed when increasing the NPDA ratio, indicating copolymerization (Figure S6c).

### Thermal Stabilities and Mechanical Strengths of the NPDA-Based PEI Films

**Figure 3:**
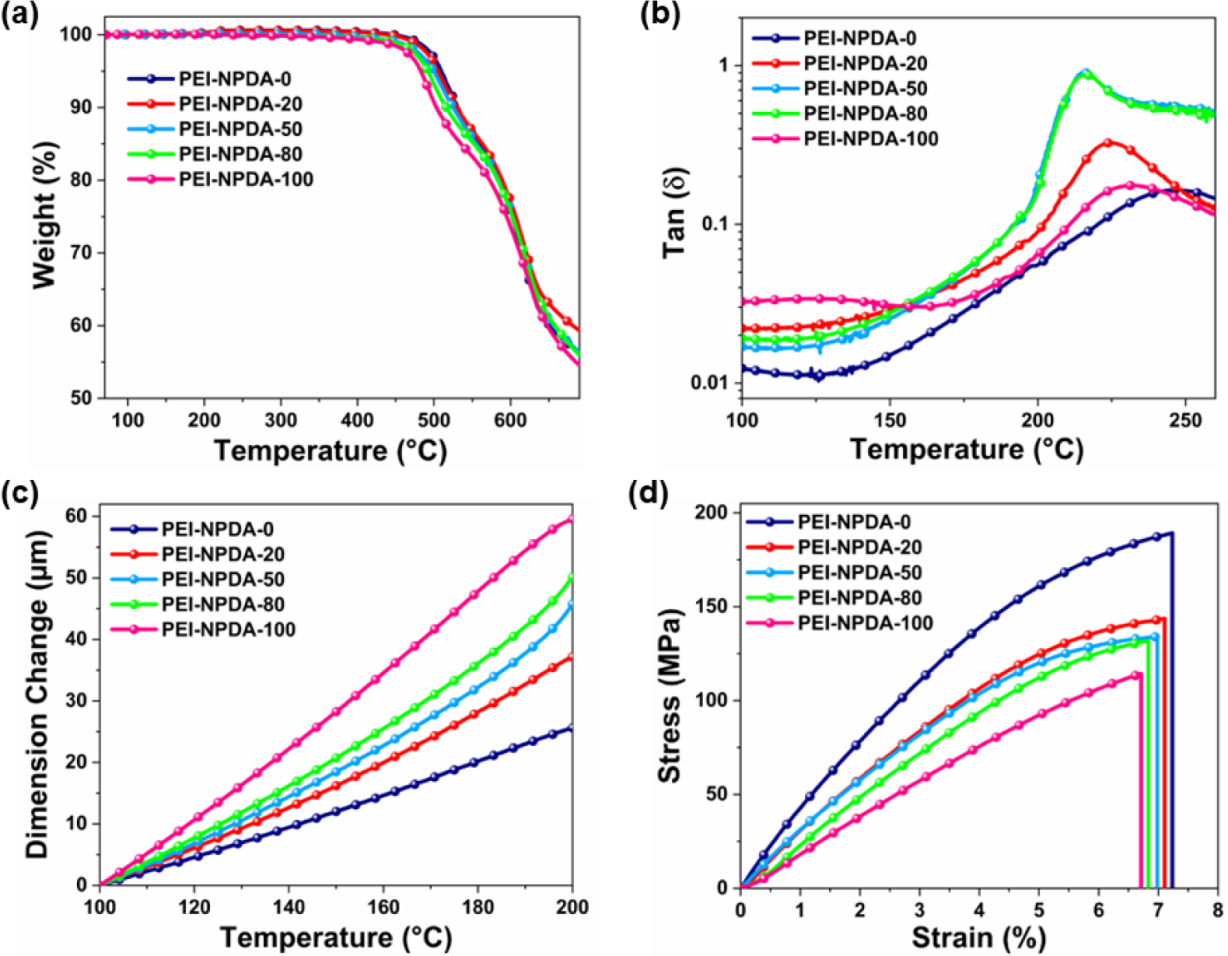
(a) TGA, (b) DMA, and (c) TMA thermograms and (d) stress–strain curves of the NPDA-based PEI films.

Thermogravimetric analysis (TGA) and dynamic mechanical analysis (DMA) are conducted to measure the thermal decomposition temperatures at 5% weight loss (*T*_d5%_) and glass transition temperature (*T*_g_) of all the NPDA-based PEI films. The resulting thermogravimetric curves are depicted in Figure 3, and the *T*_d5%_ and *T*_g_ values are summarized in Table 2. All of the PEI films show excellent thermal stabilities with *T*_d5%_ over 480 °C (Figure 3a). Among them, the PEI-NPDA-0 film shows higher thermal stability. The *T*_d5%_ values of PEI-NPDA-0, PEI-NPDA-20, PEI-NPDA-50, PEI-NPDA-80, and PEI-NPDA-100 are 509, 505, 499, 492, and 483 °C, respectively. The *T*_g_ values of all of the PEI films are calculated from the peaks of the DMA curves, as shown in Figure 3b. As outlined in Table 2, the *T*_g_ values of PEI-NPDA-0, PEI-NPDA-20, PEI-NPDA-50, PEI-NPDA-80, and PEI-NPDA-100 are 246, 224, 217, 216, and 231 °C, respectively. To evaluate the dimensional thermal stability of the NPDA-based PEIs, thermomechanical analysis (TMA) is performed to extract the coefficient of thermal expansion (CTE), as shown in Figure 3c. According to the previous reports,^9,16,18^ CTEs of PIs are highly correlated to in-plane chain orientation, which depends on the linearity and rigidity of the PI backbones. In addition, the PI films with high fluorine groups generally show high CTEs. When increasing the NPDA concentration, the CTE values of PEI-NPDA-0 to PEI-NPDA-100 gradually increase, which can be ascribed to a more balanced rigid/coplanar nature of NPDA compared with TAHQ. The CTE values of PEI-NPDA-0, PEI-NPDA-20, PEI-NPDA-50, PEI-NPDA-80, and PEI-NPDA-100 are 16.2, 23.2, 27.0, 30.0, and 38.4 ppm/°C, respectively, listed in Table 2. Furthermore, the mechanical strengths of all of the PEI films with different NPDA ratios are investigated. The stress–strain curves of all the PEI films are depicted in Figure 3d, and the results are summarized in Table 2. Generally, the mechanical properties of PIs are influenced by the rigidity and linearity of the PI backbone, aggregation, crystallinity, molecular chain interactions, and molecular weight of the PIs. In this work, all the PEI films exhibit decent mechanical strengths with maximum tensile strength within 114–190 MPa, elastic initial modulus within 2.01–3.46 GPa, and elongation at break within 6.71–7.23%. When increasing the NPDA ratio, the corresponding PEI film’s tensile strength, elastic initial modulus, and elongation at break values simultaneously decrease, which may be ascribed to the more balanced coplanar/rigid nature of the PEI backbone and molecular chain interactions.

**Table 2:** Thermal and Mechanical Properties of the NPDA-Based PEI Films

PEI films	***T*_d5%_(°C)**^a^	***T*_g_(°C)**^b^	**CTE(ppm/°C)**^c^	**σ(MPa)**^d^	***E*(GPa)**^e^	**ε(%)**^f^
**PEI-NPDA-0**	509	246	16.2	189.31	3.46	7.23
**PEI-NPDA-20**	505	224	23.2	143.72	2.64	7.10
**PEI-NPDA-50**	499	216	27.0	134.08	2.57	6.98
**PEI-NPDA-80**	492	217	30.0	131.70	2.43	6.93
**PEI-NPDA-100**	483	231	38.4	114.39	2.01	6.71

a Decomposition temperature at 5% weight loss.

b Glass transition temperature.

c Coefficient of thermal expansion.

d Tensile strength.

e Initial modulus.

f Elongation at break.

### Dielectric Properties of the NPDA-Based PEI Films

**Figure 4:**
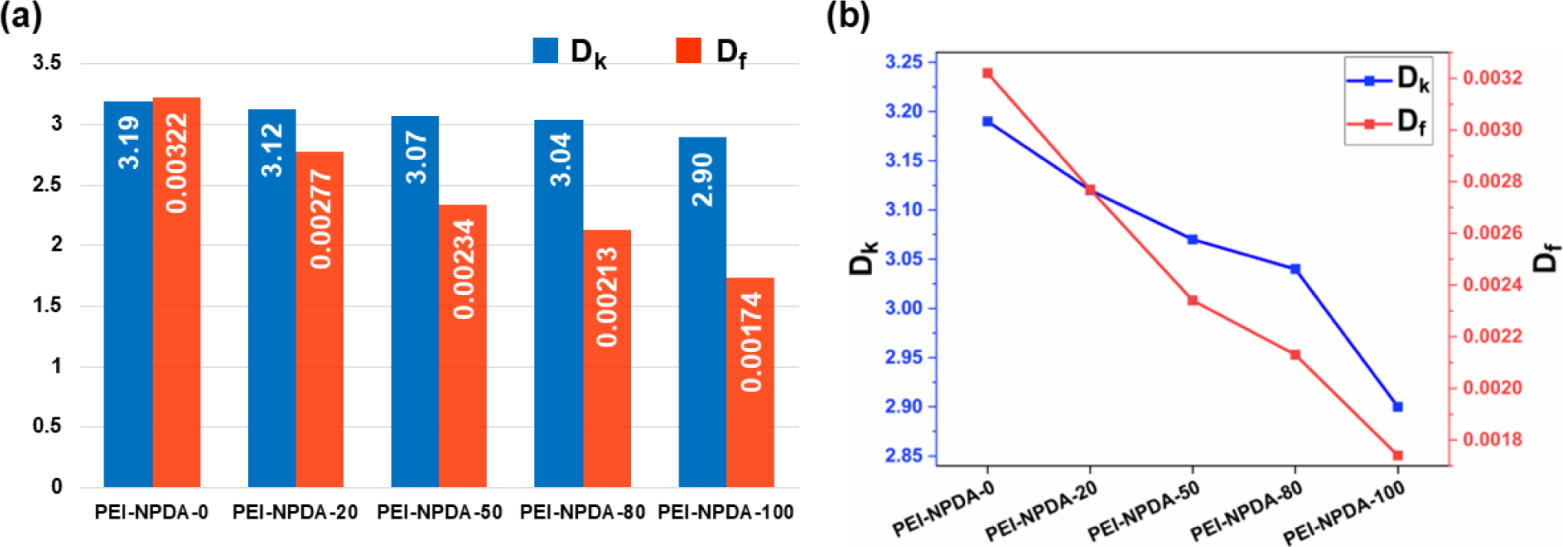
(a) Pictorial presentation of the dielectric values and (b) plots of the NPDA-based PEI films.

The low dielectric properties of polymer materials are essential for high-speed signal transmission in microelectronic devices and high-frequency communication applications.^1−5^ For achieving low dielectric values at high frequencies, there are a few structure-dielectric relationship-based strategies. For instance, Chen et al. explained the structure–property relationship to achieve low *D*_k_ and *D*_f_ values: (i) by preparing PIs without polar groups such as amide, sulfone, or ketone, (ii) by introducing ester linkages in polymer backbones that can extend the coplanarity/rigidity, (iii) by introducing fluorine groups to decrease the polarizability, and (iv) by introducing bulky backbones to achieve large molar free volume/FFV values.^9,11,16,45^ As a result of balancing rigidity/coplanarity and free volume, it is possible to obtain PIs with low *D*_k_ and *D*_f_ values at the same time. Therefore, in this study, we select the commercial fluorine group functionalized TFMB and ester-functionalized TAHQ as the primary PEI system. Subsequently, we introduce the synthesized bulky naphthalene backbone-based NPDA with an ester-functionalization monomer to the TFMB/TAHQ system. Due to the bulky backbone NPDA monomer, we suggest that the pure TFMB/NPDA system (named PEI-NPDA-100) shows more balanced rigidity/coplanarity and larger free volume compared with the pure TFMB/TAHQ system (named PEI-NPDA-0).^46−48^ Herein, we use DFT calculations to determine the molar volume and dipole moment of PEI-NPDA-0 and PEI-NPDA-100 based on one polymer monomer unit. The optimized geometries of PEI-NPDA-0 and PEI-NPDA-100 monomer units are depicted in Figure S7. From the DFT-optimized geometries, owing to the benzene ring, the PEI-NPDA-0 monomer unit shows a more linear backbone nature, higher dipole moment, and lower molar volume. On the other hand, the PEI-NPDA-100 monomer unit shows a more balanced and extended linear backbone nature, lower dipole moment, and higher molar volume because of the naphthalene core. The obtained molar volume and dipole moments are 434.63 cm^3^ and 2.56 D for PEI-NPDA-0 and 493.99 cm^3^ and 2.47 D for PEI-NPDA-100, respectively. As a consequence, when increasing the NPDA monomer ratio to the TAHQ-TFMB system, the dielectric properties of both *D*_k_ and *D*_f_ gradually decrease from 3.19 and 0.00322 for PEI-NPDA-0 to 2.90 and 0.00174 for PEI-NPDA-100, respectively. Figure 4 demonstrates the trends of the *D*_k_ and *D*_f_ values of the PEI films at 10 GHz, and the values are summarized in Table 3. For comparison, the dielectric results of the NPDA-based PEI films and recently reported PEI films are summarized in Table S1.

**Table 3:** Summary of Dielectric Properties, Density, FFV, Water Absorption, and Contact Angles of the PEI Films

**PEI films**	**D**_**k**_^a^	**D**_**f**_^b^	**V**_**molar**_^c^	**density**	**FFV**^d^	**contact angle** (deg)	**water absorption** (%)
**PEI-NPDA-0**	3.19 (±0.01)	0.00322 (±0.00005)	502.30	1.55	0.157	66.3	0.93
**PEI-NPDA-20**	3.12 (±0.01)	0.00277 (±0.00005)	540.12	1.46	0.203	67.9	0.86
**PEI-NPDA-50**	3.07 (±0.01)	0.00234 (±0.00005)	578.12	1.39	0.238	69.4	0.80
**PEI-NPDA-80**	3.04 (±0.01)	0.00213 (±0.00005)	620.16	1.32	0.272	72.9	0.76
**PEI-NPDA-100**	2.90 (±0.01)	0.00174 (±0.00005)	652.46	1.27	0.298	75.9	0.73

a Dielectric constant.

b Dissipation factor.

c Molar volume.

d Fractional free volume.

In PIs, water absorption plays a significant impact on dielectric properties due to the hydrophilic imide ring nature.^18,49^ To maintain moisture resistance and dielectric stability in humid environments, PIs should decrease their hydrophilic and water uptake characteristics. Therefore, in this study, the water contact angles and water absorption behaviors are tested to evaluate the performance of the PEI films in the presence of high humidity conditions.^50^ The water contact angle images of all of the PEI films are depicted in Figure S8, and the results are outlined in Table 3. From these results, all the PEI films exhibit a decent hydrophobic characteristic nature because the molecular packing in the fluorine-/ester-functionalized PEI films is improved. Compared with the PEI-NPDA-0 film, however, the PEI-NPDA-100 film shows an even higher hydrophobic characteristic nature, which may be due to the more balanced coplanar/rigid backbone nature in the PEI-NPDA-100 PEI films, resulting in enhanced molecular packing of the PEI-NPDA-100 PEI film. When increasing the NPDA ratio, the water absorption values show a decreasing trend from 0.93 of PEI-NPDA-0 to 0.73% of PEI-NPDA-100. The water contact angle experimental results also confirm the water absorption results. When increasing the NPDA ratio, the water contact angle shows an increasing trend from 66.3 of PEI-NPDA-0 to 75.9° of PEI-NPDA-100, which indicates weakened affinities between the film and water, resulting in decreased water absorption. The water contact angles of PEI-NPDA-0, PEI-NPDA-20, PEI-NPDA-50, PEI-NPDA-80, and PEI-NPDA-100 are 66.3, 67.8, 69.4, 72.9, and 75.9°, respectively. As a result of the good hydrophobic characteristics, PEIs exhibit stable dielectric properties in humid environments for long periods.^42,50^

### Surface Film Morphologies of the NPDA-Based PEI Films

**Figure 5:**
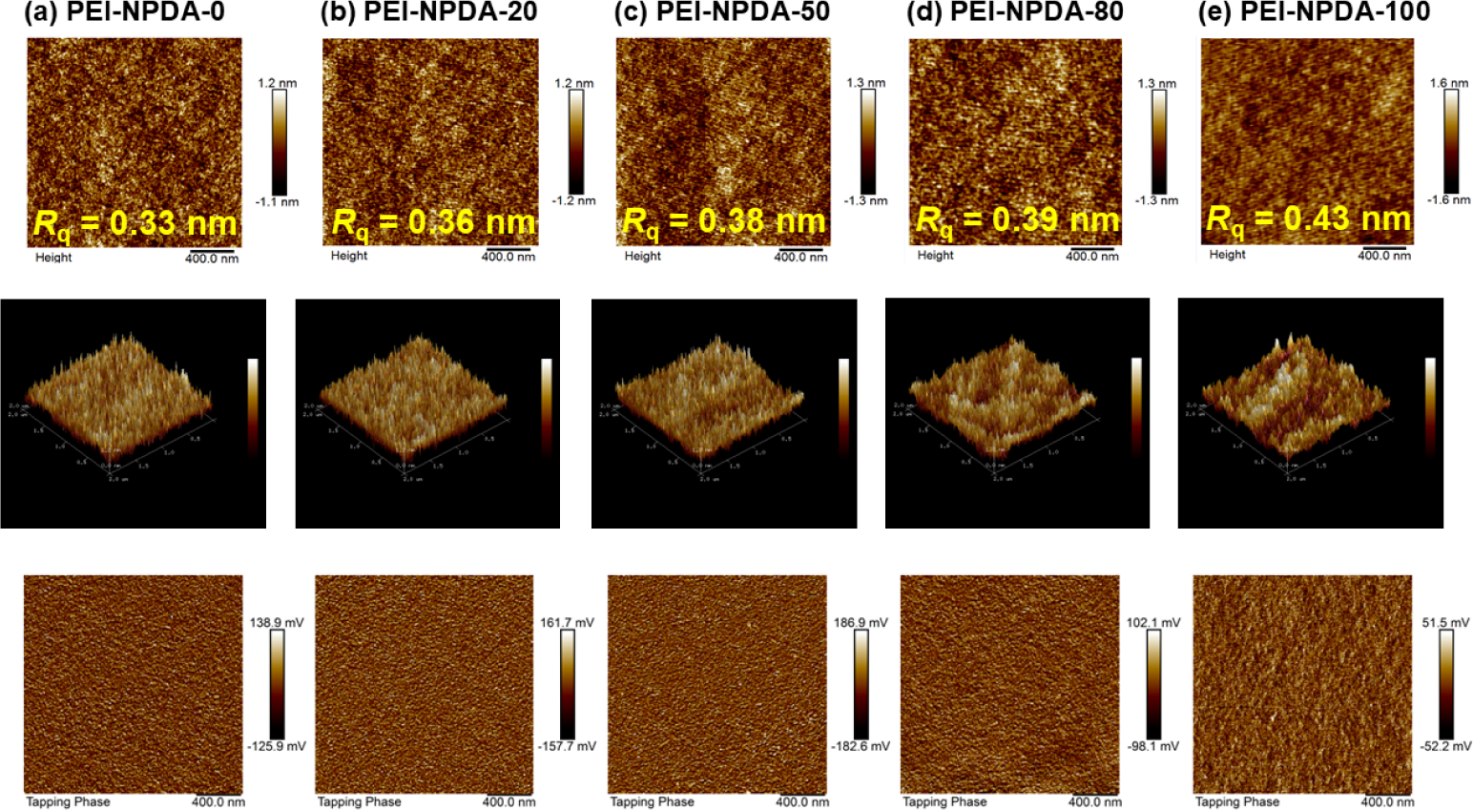
AFM height (top), three-dimensional (3D) topological (middle), and phase (bottom) images of (a) PEI-NPDA-0, (b) PEI-NPDA-20, (c) PEI-NPDA-50, (d) PEI-NPDA-80, and (e) PEI-NPDA-100 films.

To better understand the film aggregations and surface film morphologies of the NPDA-based PEI films, tapping mode atomic force microscopy (AFM) and scanning electron microscopy (SEM) analyses are conducted. For AFM analysis, all the NPDA-based PEI films are prepared by the spin-coated method on a well-dried clean glass plate, and the obtained AFM images are demonstrated in Figure 5. When increasing the concentration of NPDA dianhydride, the root-mean-square (*R*_q_) surface roughness values are gradually increased from 0.33 nm for the PEI-NPDA-0 film to 0.43 nm for the PEI-NPDA-100 film. These results indicate that the surface film morphologies of all NPDA-based PEI films are slightly aggregated and show good phase separation when increasing the NPDA ratio, which is observed in three-dimensional (3D) topological AFM images (Figure 5). The TEM images provide further insights into the differences in the morphology of the NPDA-based PEI films. As shown in Figure S9, when increasing the NPDA ratio, the phase separation and aggregation are gradually enhanced from PEI-NPDA-0 to PEI-NPDA-100 films. These SEM results are in good agreement with the AFM results.

## Conclusions

In summary, a series of PEIs with fluorine- and ester-functionalized groups are prepared using a conventional two-step process of polycondensation followed by thermal imidization. First, we construct a PEI-NPDA-0 PEI film from benzene-containing ester-functionalized TAHQ and fluorine group-functionalized TFMB monomers. Subsequently, PEI-NPDA-20, PEI-NPDA-50, PEI-NPDA-80, and PEI-NPDA-100 PEI films are prepared by the addition of 20, 50, 80, and 100% bulky naphthalene-based ester-functionalized NPDA monomers, respectively. As a result, all the PEI films exhibit excellent thermal stabilities with *T*_d5%_ over 470 °C and *T*_g_ within 215–250 °C. In addition, all the PEI films show a decent CTE in the range of 16–40 ppm/°C and good mechanical strengths. From the well-known Clausius–Mossotti equation, after the introduction of the bulky NPDA monomer, the molar free volume gradually increases, resulting in reduced dielectric values. As a consequence, the *D*_k_/*D*_f_ values gradually decrease from 3.19/0.00322 for PEI-NPDA-0 to 2.90/0.00174 for PEI-NPDA-100 under a 10 GHz frequency. Indeed, the bulky PI backbones with ester- and fluorine-based PEIs successfully reduce both dielectric (*D*_k_ and *D*_f_) properties by controlling polarizability, linearity/rigidity, and free volumes. Overall, our research results can provide practical insights and directions for the future development of structure-dielectric properties.

## Supporting Information Available

^1^H and ^13^C NMR spectra, LR-FD mass spectra, ATR-FT-IR data, DFT calculation results, dielectric properties, contact angle images, and SEM images (PDF)
