# Supplementary material for: Achieving Low Dissipation Factors and Low Dielectric Constants via Thermally Stable Naphthalene-Based Poly(ester-imide)s with Fluorine Groups
Source: ACS Appl Mater Interfaces. 2025 Mar 13;17(12):18931–9. doi: 10.1021/acsami.5c00599 (PMC11956007; doi:10.1021/acsami.5c00599)
Supplement: Supplementary file 1 — am5c00599_si_001.pdf [file am5c00599_si_001.pdf]

Supporting Information

Achieving Low Dissipation Factors and Low Dielectric Constants via Thermally Stable Naphthalene-Based Poly(ester-imide)s with Fluorine Groups

Manohar Reddy Busireddy,[#,a,b] Ling-Huan Meng,[#,a,b] Jin-Wei Lin,[a,b] Wei-Chung Ke,[a,b] Jiun-Tai Chen,[a,b] and Chain-Shu Hsu[*,a,b]

[a]*Department of Applied Chemistry, National Yang Ming Chiao Tung University, 1001 University Road, Hsinchu 300093, Taiwan E-mail: cshsu@nycu.edu.tw*

[b]*Center for Emergent Functional Matter Science, National Yang Ming Chiao Tung University, 1001 University Road, Hsinchu 300093, Taiwan*

Note:

# = Both authors contributed equally to this work.

S. No.	TABLE OF CONTENTS	Page No.
**1.**	NMR and Mass Spectral Data Analysis	**S2**
**2.**	ATR-FT-IR Spectral Data Analysis	**S5**
**3.**	DFT Calculations	**S5**
**4.**	Dielectric Properties	**S6**
**5.**	Contact Angle Measurements	**S7**
**6.**	SEM Measurements	**S7**
**7.**	References	**S8**

1. NMR and Mass Spectral Data Analysis

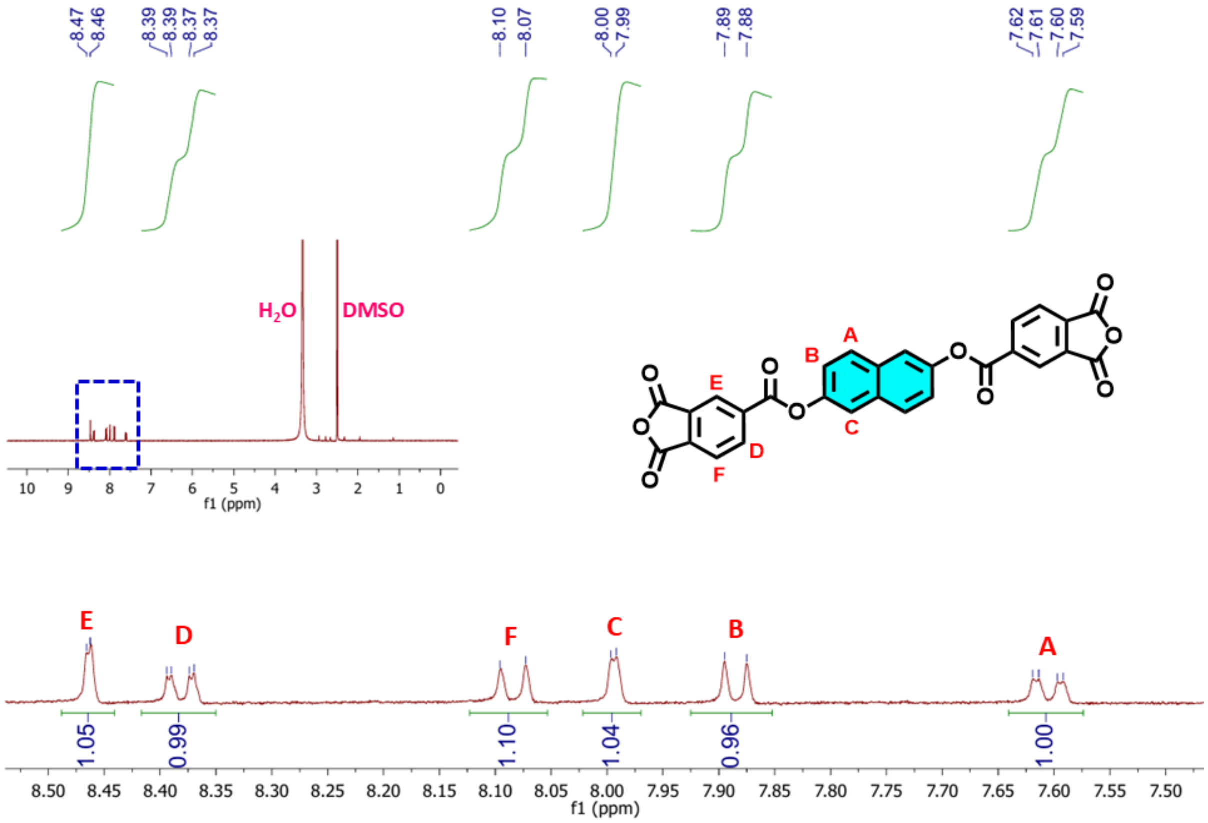

Figure S1. ^{1}H NMR spectrum of NPDA monomer.

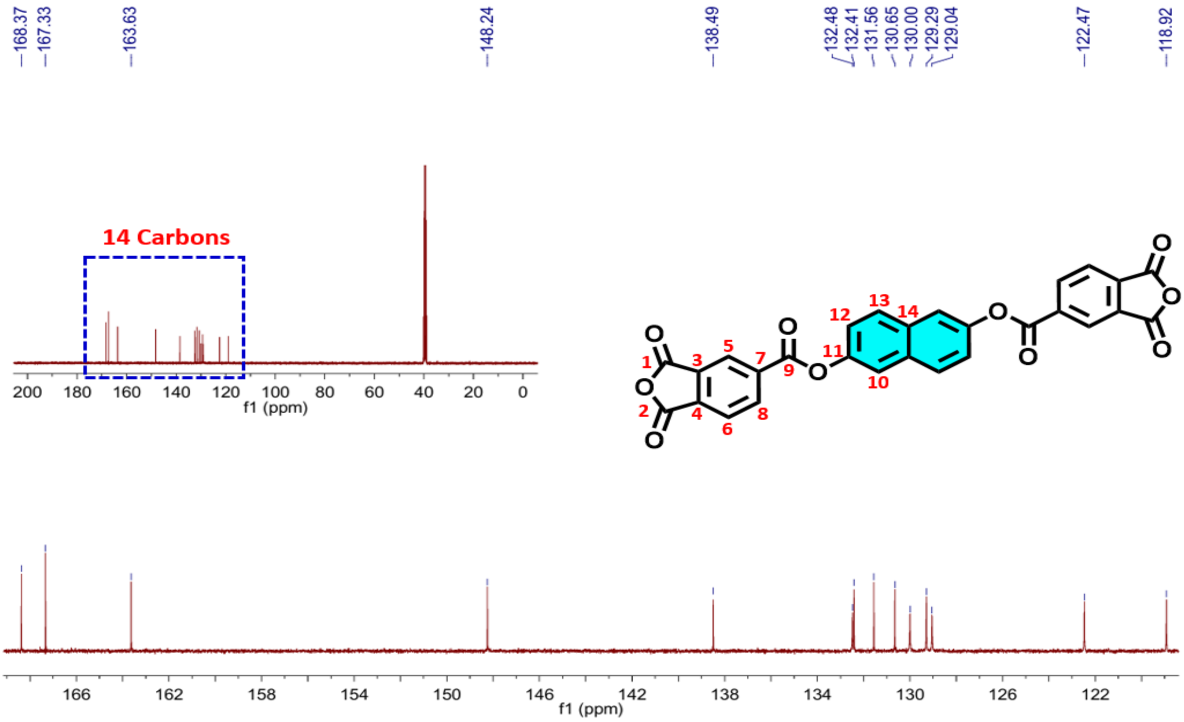

Figure S2. ^{13}C NMR spectrum of NPDA monomer.

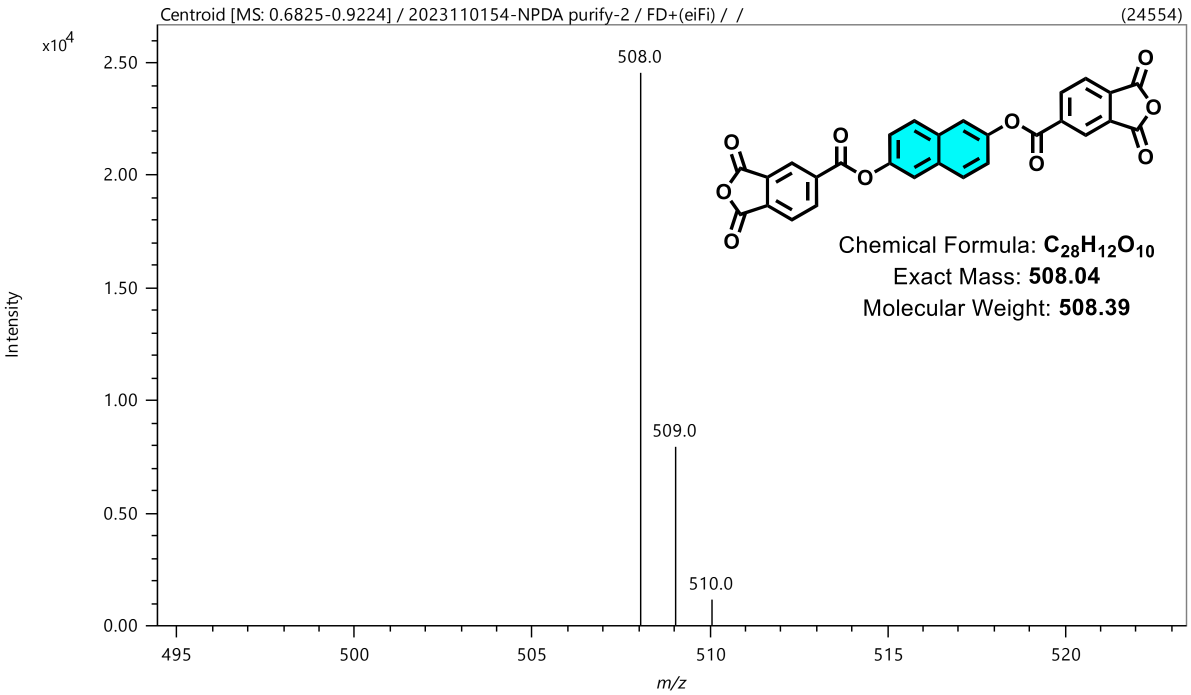

Figure S3. FD-MS spectrum of NPDA monomer.

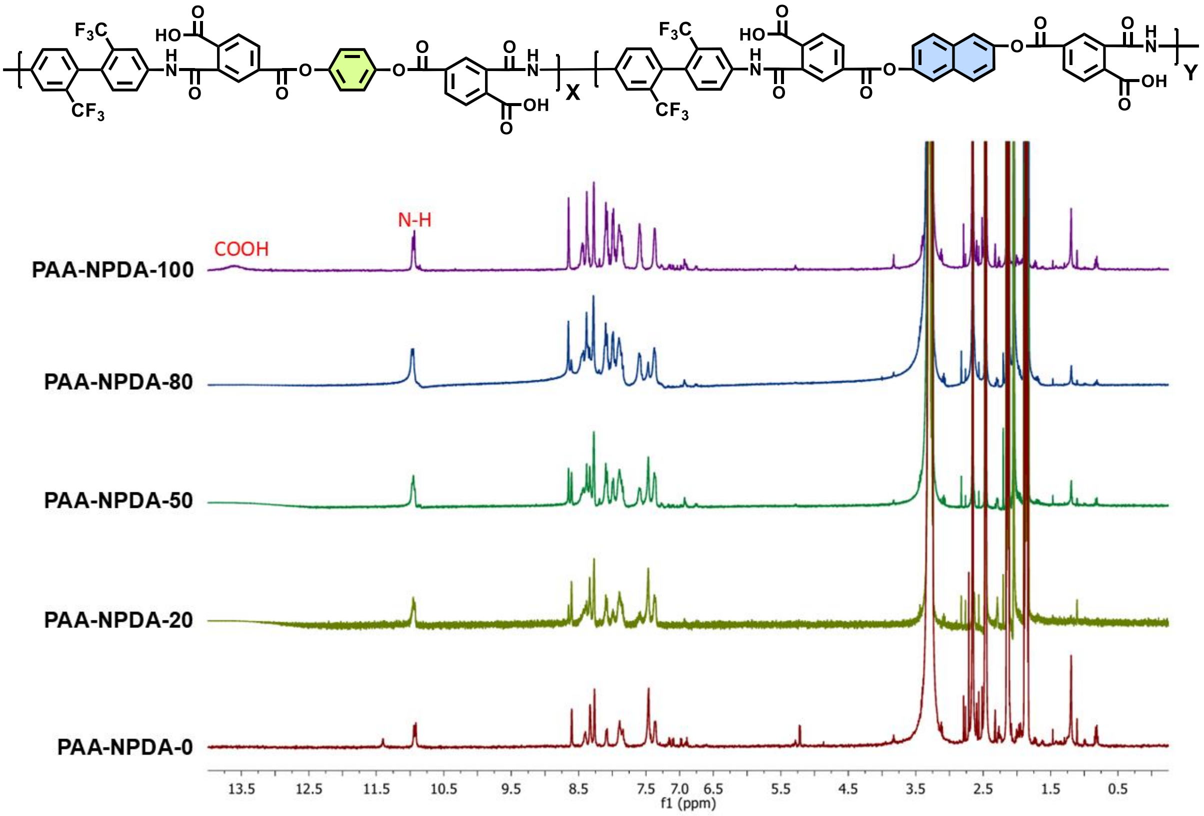

Figure S4. ^{1}H NMR spectra of all NPDA-based PAA solutions at different weight ratios of NPDA from 0–100%.

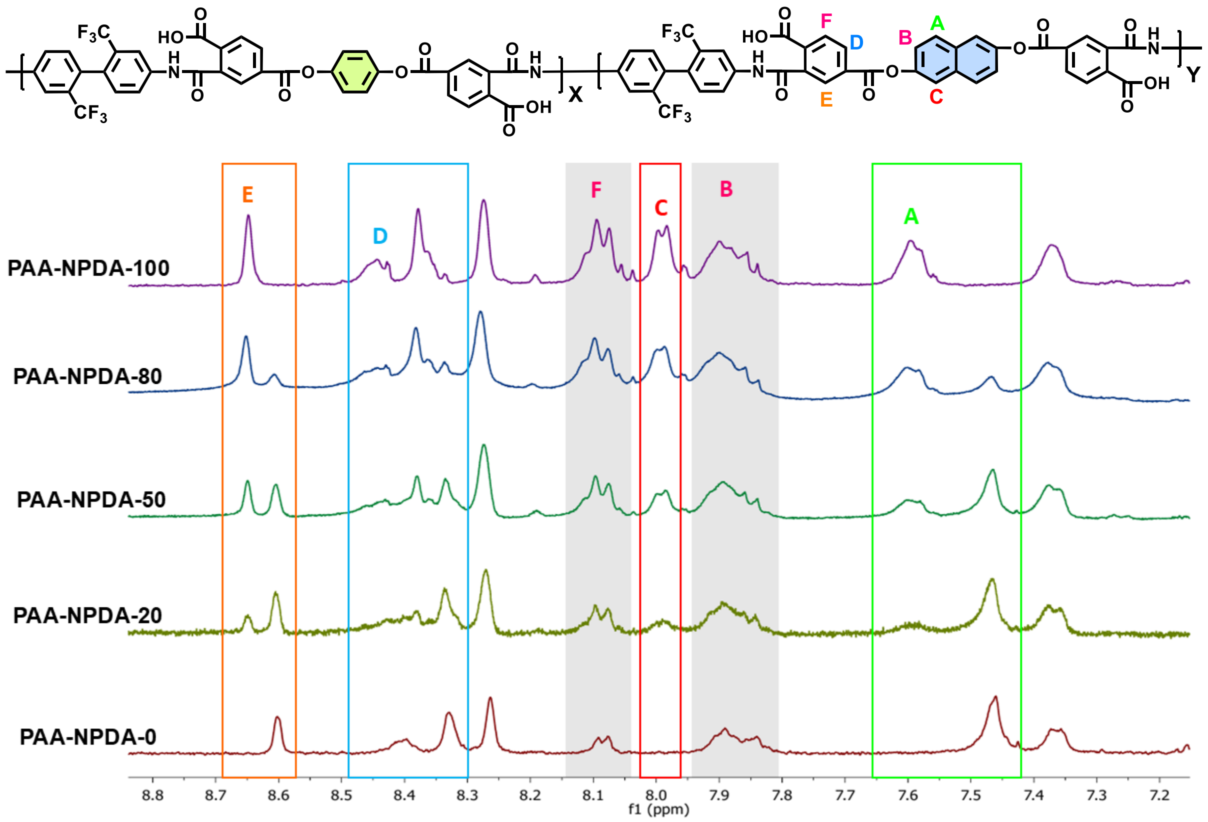

Figure S5. Expanded ^{1}H NMR spectra of all NPDA-based PAA solutions at different weight ratios of NPDA from 0–100%.

2. ATR-FT-IR Spectral Data Analysis

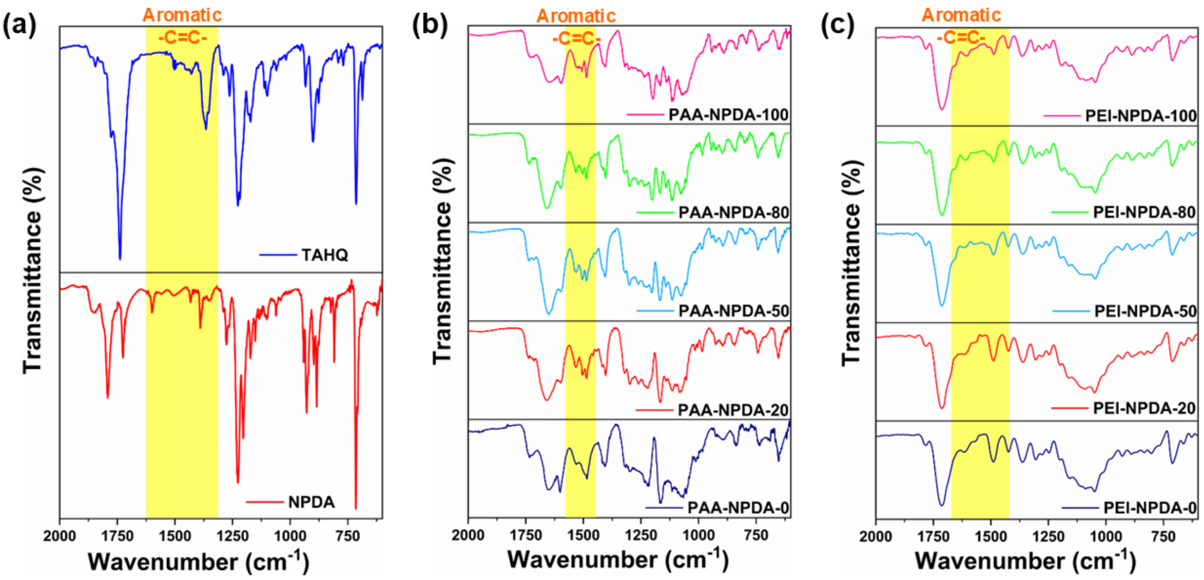

Figure S6. ATR-FT-IR spectra of the (a) TAHQ/NPDA monomers, (b) NPDA-based PAA solutions, and (c) NPDA-based PEIs.

3. DFT Calculations

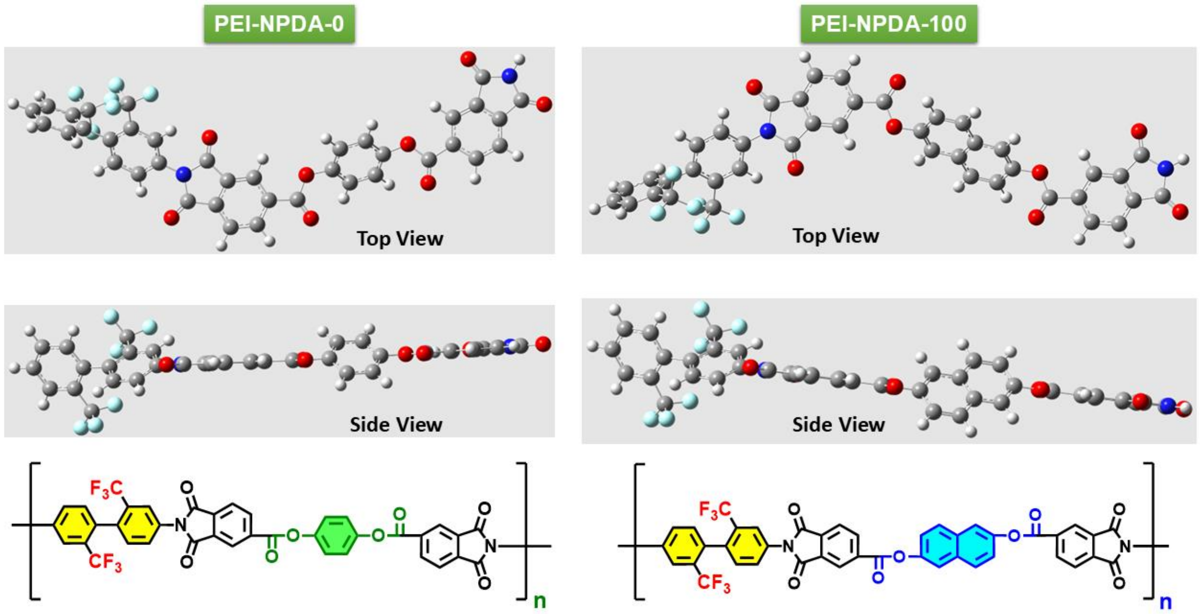

Figure S7. DFT optimized geometries of PEI-NPDA-0 and PEI-NPDA-100 PEI monomer units.

4. Dielectric Properties

Table S1. Summarized dielectric constant/dissipation factor values of a few recently reported poly(ester- imide)s (PEIs) at 10–38 GHz frequency

PEIs	Frequency (GHz)	Dielectric Properties		References
		D_k [(a)]	D_f [(b)]	
TAHQ/ABHQ	10	3.03	0.0019	1
TAHQ/BAEHF	10	2.67	0.0036	1
TAHQ/BAPHF	10	2.44	0.0028	1
TAHQ/ABHQ	10/38	3.20/3.21	0.0028/0.0033	2
TAHQ/ABHQ+TEB	10/38	2.86/2.94	0.0031/0.0027	2
A2EB-ODA-TA2EN	10	3.24	0.0015	3
TAHQ/ABHQ+M10	10/38	2.90/2.64	0.0017/0.0020	4
TAHQ/ABHQ+M20	10/38	2.77/2.53	0.0018/0.0025	4
s-BPDA/AB-MeOHQ	10	3.18	0.00314	5
TA-26NAHB/4,4′-ODA	10	3.01	0.00206	6
PI-mmm-T	10	~3.2	~0.00287	7
A2EB-ODPA	10	3.34	0.0015	8
ODA-TA2EB	10	3.32	0.0018	8
BAHQ-TFMB	10	3.24	0.00169	9
TAHQ/ODA	10	3.46	0.0038	10
TAHQ/APAB	10	3.64	0.0040	10
AXEB/TA2EB	10	3.25	0.0013	11
AXEB/6FDA	10	~2.80	0.0030	11
ISS/TFDB	10	2.96	0.0092	12
CoPI (ISS_9-ISM_1)	10	2.96	0.00944	12
CoPI (ISS_7-ISM_3)	10	2.92	0.00952	12
**PEI-NPDA-0**	**10**	**3.19**	**0.00322**	**This work**
**PEI-NPDA-20**	**10**	**3.12**	**0.00277**	**This work**
**PEI-NPDA-50**	**10**	**3.07**	**0.00234**	**This work**
**PEI-NPDA-80**	**10**	**3.04**	**0.00213**	**This work**
**PEI-NPDA-100**	**10**	**2.90**	**0.00174**	**This work**

[(a)] Dielectric Constant. [(b)] Dissipation Factor.

5. Contact Angle Measurements

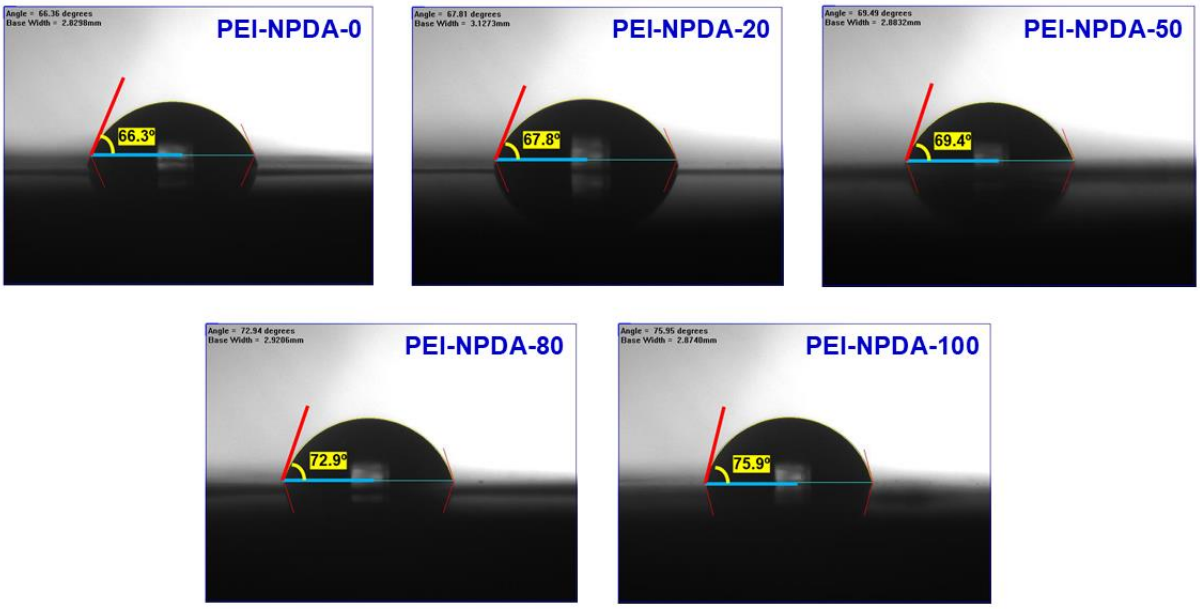

Figure S8. Contact angle images of the NPDA-based PEI films.

6. SEM Measurements

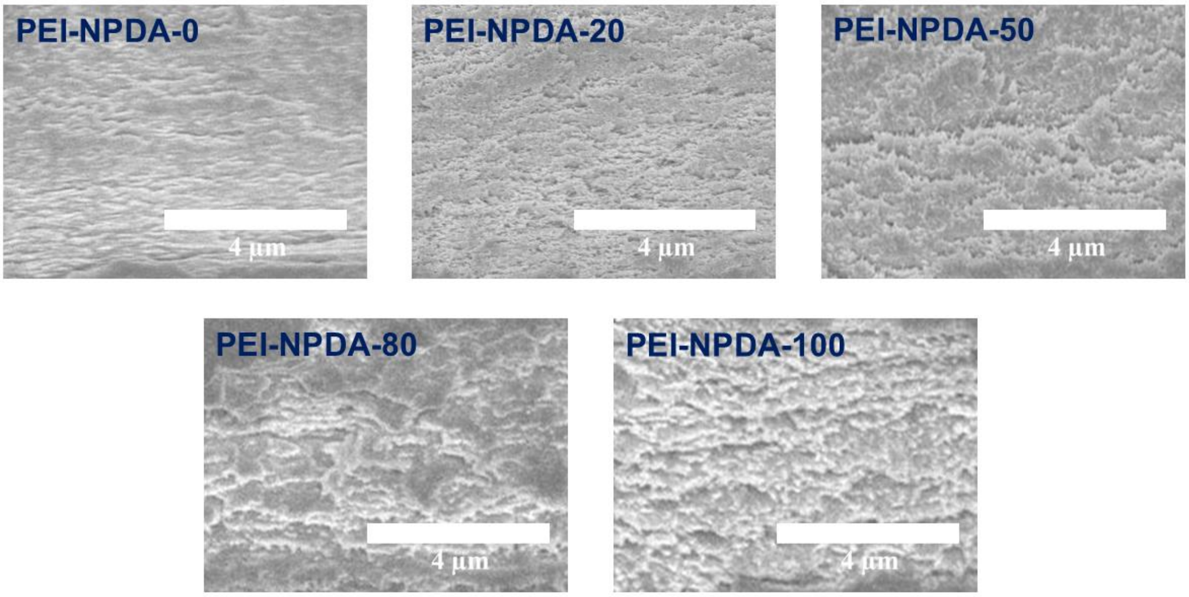

Figure S9. SEM images of the PEI-NPDA-0, PEI-NPDA-20, PEI-NPDA-50, PEI-NPDA-80, and PEI-NPDA-100 films.

7. References

1. Chen, Y. C.; Lin, Y. C.; Chang, C. C.; Kuo, M.; Ueda, W. C.; Chen, W.-C. Investigation of the Structure-Dielectric Relationship of Polyimides with Ultralow Dielectric Constant and Dissipation Factors Using Density Functional Theory. *Polymer* **2022**, *256*, 125184.
2. Cheng, Y.-C.; Chen, Y.-C.; Lin, Y.; Kuo, C.-C.; Chen, W. Exploring the Cross-Linking Effect on Decreasing the Dielectric Constant and Dissipation Factor of Poly(ester imide)s at a High Frequency of 10–40 GHz. *ACS Appl. Polym. Mater.* **2023**, *5*, 7907–7917.
3. Zhang, C.; He, X.; Lu, Q. The Structure Design of Poly(ester imide)s with Low Dielectric Loss and High Mechanical Properties. *J Mater. Chem. C* **2025**, DOI: https://doi.org/10.1039/d4tc04308a.
4. Mi, M. C.; Szu, F. E.; Cheng, Y. C.; Tsai, C. H.; Chen, J. H.; Huang, J. H.; Kuo, C. C.; Lin, Y. C.; Leung, M. K.; Chen, W. C. Semiaromatic Poly (ester imide) Copolymers with Alicyclic Diamines for Low-K Properties at a High Frequency of 10–40 GHz. *ACS Appl. Polym. Mater.* **2024**, *6*, 11137–11148.
5. Hasegawa, M.; Hishiki, T. Poly(ester imide)s Possessing Low Coefficients of Thermal Expansion and Low Water Absorption (V). Effects of Ester-Linked Diamines with Different Lengths and Substituents. *Polymers* **2020**, *12*, 859.
6. Hasegawa, M.; Fukuda, T.; Ishii, J. Poly(ester imide)s with Low Linear Coefficients of Thermal Expansion and Low Water Uptake (VII): A Strategy to Achieve Ultra-Low Dissipation Factors at 10 GHz. *Polymers* **2024**, *16*, 653.
7. Li, Y. D.; Li, H.; Feng, L. K.; Bao, F.; Wang, M. L.; Zhu, C. Z.; Zheng, Z. H.; Ding, X. B.; Xu, J. Molecular Chain Flexibility and Dielectric Loss at High-Frequency: Impact

of Ester Bond Arrangement in Poly(ester imide)s. *Chinese J. Polym. Sci.* **2024**, *42*, 1122–1133.

8. Zhang, C.; He, X.; Lu, Q. High-Frequency Low-Dielectric-Loss in Linear-Backbone-Structured Polyimides with Ester Groups and Ether Bonds. *Commun. Mater.* **2024**, *5*, 55.
9. Qin, Y.; Yin, Q.; Lyu, J.; Wang, X.; Liu, X. Preparation of polyimide films with ultralow dielectric loss at high frequency by reducing intermolecular friction. *Polymer* **2024**, *309*, 127432.
10. Kuo, C.-C.; Lin, Y.-C.; Chen, Y.-C.; Wu, P.-H.; Ando, S.; Ueda, M.; Chen, W.-C. Correlating the Molecular Structure of Polyimides with the Dielectric Constant and Dissipation Factor at a High Frequency of 10 GHz. *ACS Appl. Polym. Mater*. **2021**, *3*, 362–371.
11. Zhang, C.; He, X.; Lu, Q. Polyimide Films with Ultralow Dielectric Loss for 5G Applications: Influence and Mechanism of Ester Groups in Molecular Chains. *Eur. Polym. J.* **2023**, *200*, 112544.
12. Sawada, R.; Ando, S. Polarization Analysis and Humidity Dependence of Dielectric Properties of Aromatic and Semialicyclic Polyimides Measured at 10 GHz. *J. Phys. Chem. C* **2024**, *128*, 6979–6990.
